# Effect of constraint-induced movement therapy combined with neuromuscular electrical stimulation on upper extremity function in stroke survivors: A protocol for systematic review

**DOI:** 10.1097/MD.0000000000034249

**Published:** 2023-07-14

**Authors:** Mahmoud M. Dboba, Nor Azlin Mohd Nordin, Haidzir Manaf, Hanif Farhan Mohd Rasdi

**Affiliations:** a Physiotherapy Program, Centre for Rehabilitation and Special Needs Studies, Faculty of Health Sciences, Universiti Kebangsaan Malaysia, Kuala Lumpur, Malaysia; b Centre for Physiotherapy Studies, Faculty of Health Sciences, Universiti Teknologi MARA, Selangor, Malaysia; c Occupational Therapy Program, Center for Rehabilitation and Special Needs Studies, Faculty of Health Sciences, Universiti Kebangsaan Malaysia, Kuala Lumpur, Malaysia.

**Keywords:** constraint-induced movement therapy, neuromuscular electrical stimulation, stroke, upper extremity

## Abstract

Motor impairment is common after a stroke and directly affects the function and quality of life of stroke survivors. Constraint-induced movement therapy and neuromuscular electrical stimulation are interventions that facilitate functional recovery of the upper extremities of a particular subgroup of stroke survivors. The objective of this study was to summarize the available evidence on the effects of neuromuscular electrical stimulation combined with constraint-induced movement therapy in patients with stroke.

**Methods:**

We conducted a comprehensive search of published articles in electronic databases, including PubMed, Scopus, PEDro, Medline (via Ovid), EMBASE, Cochrane Library, and Web of Science, using the following search terms: “stroke”; “upper extremity”; “Constraint-Induced Movement Therapy”; and “Neuromuscular Electrical Stimulation.” The search included published studies, conferences, and presentations. The article selection, data extraction, and quality evaluation will be conducted independently by 2 reviewers. The 3rd and 4th reviewers will assist in resolving any disagreements that may arise between the 2 reviewers. The risk of bias in the included studies will be assessed using the PEDro scale and Cochrane risk of bias assessment tool. Narrative synthesis and meta-analysis will be performed based on the characteristics of the included articles, including the risk of bias (if sufficient information is available).

**Results:**

This review summarizes the available evidence and could assist therapists in choosing the best treatment for poststroke upper extremity dysfunction.

**Conclusion:**

This study will provide the available evidence on the effectiveness of CIMT and NMES on upper extremity function in patients with stroke.

**Ethics and dissemination:**

Ethical approval is not required because the review will be based on publicly available literature. The findings of this study will be published in a peer-reviewed journal, and updates will be made depending on whether sufficient additional evidence modifies the conclusions of the review. Any changes made to the methods throughout the review will be stated in the article.

**Systematic review registration:**

PROSPERO CRD42023415645.

## 1. Introduction

Stroke is a significant health problem and cause of disability in adults worldwide.^[1]^ According to previous reports, there were approximately 101 million prevalent cases of stroke in 2019 and 143 million disability-adjusted life years due to stroke.^[2,3]^

Stroke is one of the main causes of acquired disabilities in adults.^[4,5]^ Approximately 80% of survivors have motor impairments in their upper extremities^[6]^ that gravely affect their ability to perform activities of daily living and social participation.^[7]^ Upper extremity impairment is one of the most common motor defects after a stroke.^[8]^ Approximately 30% to 66% of stroke survivors report persistent movement impairment of their affected arm in daily life,^[9]^ and 15% to 30% of stroke survivors experience long-lasting hemiparesis in the affected arm.^[10]^

Improving upper extremity function is a core element of stroke rehabilitation, which is needed to maximize patient outcomes and reduce disability.^[11,12]^ Constraint-induced movement therapy (CIMT) has been widely used for upper extremity rehabilitation following stroke, especially in laboratories and clinics. CIMT is a rehabilitation approach that improves motor function impairments after stroke.^[13]^ Neurorehabilitation studies have shown that CIMT can increase both motor function and the use of the paretic arm in adult patients after stroke,^[14–16]^ and that these improvements parallel changes in the activation of the brain sensorimotor network.^[17,18]^

Neuromuscular electrical stimulation (NMES) is widely used in stroke rehabilitation for motor impairments by improving and assisting volitional movements.^[19]^ Given the common motor impairments following stroke, NMES-based interventions aimed at improving motor function and quality of life should ideally be able to contract weak muscles along the affected limb to address paresis, facilitate individual joint control and coordination to restore fractionated movements and reduce abnormal synergistic patterns and hypertonia.^[20]^

Several studies have indicated that NMES significantly affects limb rehabilitation in patients with stroke. Some studies have reported that electrical stimulation therapy positively relieves shoulder pain and improves muscle function in patients with stroke.^[21,22]^ Its main advantage depends on the noninvasive regulation of brain function and its strong spatial localization ability, which provides broad room for treating further brain functional regions.^[23,24]^

CIMT and NMES are used to counteract learned nonuse phenomena and improve motor function and quality of life following a stroke. These findings raise an important clinical question regarding rehabilitation therapies: what is the effect of CIMT and NMES on improving poststroke upper extremity function? Do these combined interventions yield better results than other therapies? Thus, to help physiotherapists and occupational therapists make better choices for improving upper extremity function, a systematic review should be conducted to summarize the evidence of various rehabilitation therapies and identify the most effective therapy for poststroke upper limb impairment. Therefore, this study aimed to summarize the available evidence on the effects of combining NMES with CIMT in patients with stroke and determine the impact of CIMT and NMES on improving poststroke upper extremity function. This review also provides treatment options based on their effectiveness.

## 2. Methods

### 2.1. Study protocol and registration

This systematic review protocol will be reported in accordance with the Preferred Reporting Items for Systematic Review and Meta-analysis Protocol Guidelines (PRISMA-P). The protocol has been registered in the PROSPERO database (CRD42023415645). Any emendation of this protocol will be updated on the PROSPERO registration. The findings of this study will be published publicly.

### 2.2. Eligibility criteria

The inclusion criteria in this study were designed using the 4 key elements of the study design, participants, interventions, and outcome measurements.

#### 2.2.1. Type of studies.

The studies included in this systematic review will include randomized controlled trials, experimental designs (pretest and posttest), and any other study designs, except for systematic reviews and meta-analyses.

#### 2.2.2. Types of participants.

We will include studies that involve adult human patients (over 18 years old) of either sex or ethnicity after the first or recurrent stroke.

#### 2.2.3. Types of interventions.

We will select all trials assessing CIMT along with NMES on upper extremity function among stroke patients compared with any other treatment or no treatment.

#### 2.2.4. Outcome measurements.

The primary outcome focused on motor function. Secondary outcomes will include motor impairment, activities of daily living, self-efficacy, and quality of life, as reported in previous studies.

### 2.3. Exclusion criteria

The following will be excluded: systematic reviews and meta-analyses, duplicate or unextracted data, and studies without access to the full text.

### 2.4. Article sources and search strategy

Electronic databases will be searched using strategies designed based on the requirements of each database, including the combination of controlled vocabulary and search terms related to the research question and inclusion criteria. The validated filters will also be used to assist in retrieving only the appropriate studies. The following databases will be searched from their earliest dates to the most recent dates: (PubMed, Web of Science, Cochrane Library, PEDro, EMBASE, Scopus, and CENTRAL). In addition, we will search the reference lists of relevant articles to identify further studies and follow those that cite pertinent studies.

The strategy of the search will involve 3 kinds of terms: “stroke,” “upper extremity,” “Constraint-Induced Movement Therapy,” and “Neuromuscular Electrical Stimulation.” The search process is summarized in Table 1. All published studies, conferences, and presentations will be searched.

**Table 1:** Search strategy used in electronic databases.

Number	Search items
**#1**	(Constraint-Induced Movement Therapy OR Constraint Induced Movement Therapy OR CIMT OR Constraint Induced Therapy OR CIT OR modified Constraint-Induced Therapy OR modified Constraint Therapy OR mCIMT OR forced use OR forced-use)
**#2**	(Neuromuscular Electrical Stimulation OR NMES OR NMS OR Electromyography-triggered neuromuscular stimulation OR EMG-stim OR ETMS OR Electrical stimulation)
**#3**	(Cerebrovascular accident OR stroke OR poststroke OR post stroke OR brain injury)
**#4**	(Upper Extremity OR UE OR Upper limb)
**#5**	#1 AND #2 AND #3 AND #4 AND NOT #5

### 2.5. Searching other resources

Additionally, we will explore the reference lists of the included articles identified throughout the screening for any potentially pertinent studies. Grey literature, which comprises conference proceedings, will also be searched.

### 2.6. Study selection process

**Figure 1.**
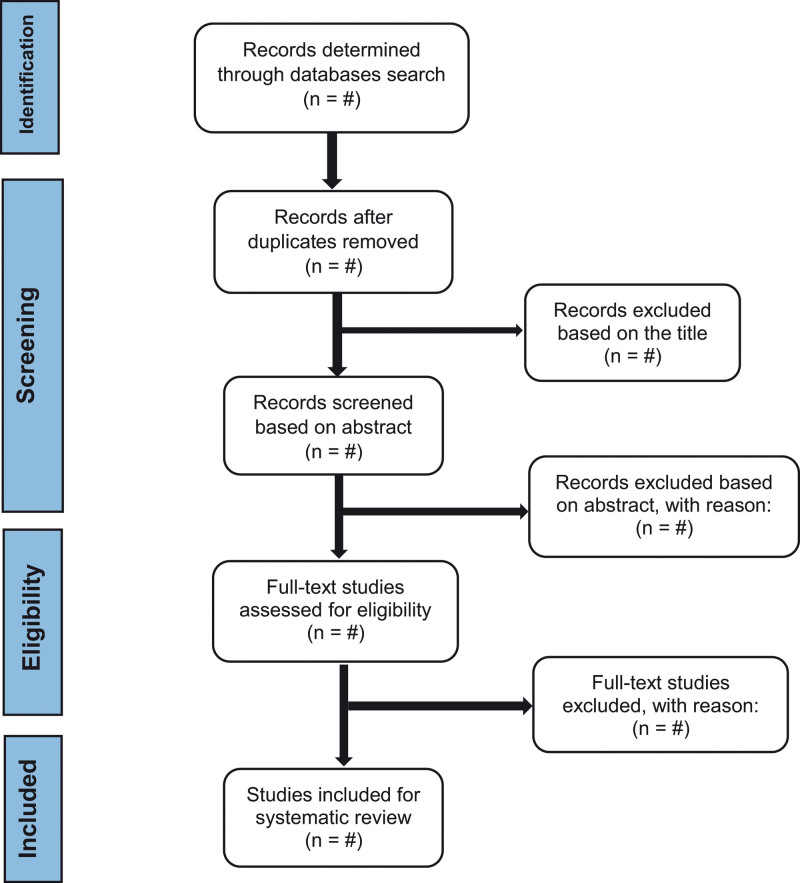
PRISMA flowcharts of the study selection process.

Following the removal of duplicates from the studies, the list of identified articles will first be checked for its title, followed by its abstract. Subsequently, full-text studies will be retrieved and investigated to obtain the final list of eligible studies. Study selection will be performed based on the PRISMA flow chart for systematic reviews and meta-analyses (see Fig. 1).

### 2.7. Data extraction

The data will be extracted independently by 2 authors. Both authors must come to an agreement. In cases of disagreement, 2 additional authors must be reached by consensus. In cases where the data are unclear or missing, the authors of the included research will be contacted. The following sections are included in the data extraction.

1. Study design.
2. Population details: population age, size, sex, side of paresis, time since stroke, unilateral or bilateral stroke, and first-ever or recurrent stroke.
3. Methodological quality of trials: details of the random process, dropout, blinding, reporting, etc.
4. The treatment program included each treatment’s overall duration, aims, and most important characteristics.
5. Outcome measurements.

### 2.8. Data analysis and assessment of risk of bias

Narrative synthesis and meta-analysis will be conducted based on the characteristics of the included articles, including the risk of bias (if sufficient information is available).

Two authors will independently assess the risk of bias for each included study, meeting the criteria provided in the Cochrane Handbook for Systematic Reviews of Interventions.^[25]^ Any disagreements in the assessment will be resolved by additional discussion or by involving 2 more authors. The following domains will be assessed for risk of bias:

1. Random sequence generation.
2. Allocation concealment.
3. Blinding of investigators and participants.
4. Blinding of the outcome assessment.
5. Incomplete outcome data.
6. Selective outcome reports.
7. Other bias.

For each domain, the risk of bias will be rated as low, high, or undetermined. In the “Risk of bias” tables, information from the study reports will be provided with a rationale for our judgment.

## 3. Discussion

Stroke is one of the most common neurological diseases that lead to disability in the elderly population and functional impairment of the upper extremity,^[26]^ which affects the performance of activities in daily life.^[27]^ Stroke is a major cause of long-term disability.^[28]^

To achieve greater functional recovery of the upper extremities following stroke onset, motor rehabilitation techniques such as CIMT may need to be combined with sensorimotor stimulation techniques such as NMES. This may help recruit more neuronal areas of the brain, which will help to control motor function.^[29]^

Several systematic reviews and meta-analyses have been conducted to investigate the efficacy of CIMT and to compare it with numerous rehabilitation therapies. To date, no systematic review has been conducted to assess the combined efficacy of CIMT and NMES on the upper extremities in stroke survivors compared to other rehabilitation therapies. Therefore, this review is necessary to determine the comparative effects of these rehabilitation therapies. To the best of our knowledge, this is the first systematic review to investigate the effects of CIMT and NMES on upper extremity function in stroke survivors.

This systematic review could help therapists choose the best treatment for poststroke upper extremity dysfunction. Moreover, we hope this review’s results will provide evidence for future guidelines or recommendations related to poststroke upper extremity rehabilitation. In addition, these results will contribute to improving the rehabilitation methods for stroke survivors.

## Author contributions

**Conceptualization:** Mahmoud M. Dboba, Nor Azlin Mohd Nordin, Haidzir Manaf, Hanif Farhan Mohd Rasdi.

**Data curation:** Mahmoud M. Dboba.

**Formal analysis:** Mahmoud M. Dboba.

**Funding acquisition:** Mahmoud M. Dboba.

**Investigation:** Mahmoud M. Dboba, Haidzir Manaf.

**Methodology:** Mahmoud M. Dboba, Nor Azlin Mohd Nordin, Haidzir Manaf, Hanif Farhan Mohd Rasdi.

**Project administration:** Nor Azlin Mohd Nordin.

**Resources:** Mahmoud M. Dboba.

**Software:** Mahmoud M. Dboba.

**Supervision:** Nor Azlin Mohd Nordin, Haidzir Manaf, Hanif Farhan Mohd Rasdi.

**Validation:** Nor Azlin Mohd Nordin, Haidzir Manaf, Hanif Farhan Mohd Rasdi.

**Visualization:** Mahmoud M. Dboba, Nor Azlin Mohd Nordin, Haidzir Manaf, Hanif Farhan Mohd Rasdi.

**Writing – original draft:** Mahmoud M. Dboba.

**Writing – review & editing:** Mahmoud M. Dboba, Nor Azlin Mohd Nordin, Haidzir Manaf, Hanif Farhan Mohd Rasdi.
